# TMB in NSCLC: A Broken Dream?

**DOI:** 10.3390/ijms22126536

**Published:** 2021-06-18

**Authors:** Sara Bravaccini, Giuseppe Bronte, Paola Ulivi

**Affiliations:** IRCCS Istituto Romagnolo per lo Studio dei Tumori (IRST) “Dino Amadori”, 47014 Meldola, Italy; sara.bravaccini@irst.emr.it (S.B.); paola.ulivi@irst.emr.it (P.U.)

**Keywords:** TMB, NSCLC, immune checkpoint inhibitors

## Abstract

Although immune checkpoint inhibitors have changed the treatment paradigm of a variety of cancers, including non-small-cell lung cancer, not all patients respond to immunotherapy in the same way. Predictive biomarkers for patient selection are thus needed. Tumor mutation burden (TMB), defined as the total number of somatic/acquired mutations per coding area of a tumor genome (Mut/Mb), has emerged as a potential predictive biomarker of response to immune checkpoint inhibitors. We found that the limited use of TMB in clinical practice is due to the difficulty in its detection and compounded by several different biological, methodological and economic issues. The incorporation of both TMB and PD-L1 expression or other biomarkers into multivariable predictive models could result in greater predictive power.

## 1. Introduction

### 1.1. Etiopathogenesis of Lung Cancer

Lung cancer is the most common cause of death from cancer worldwide, with only 30–40% of tumors diagnosed at an early and resectable stage of disease [1]. Non-small-cell lung carcinoma (NSCLC) is any type of epithelial lung cancer other than small-cell lung carcinoma (SCLC).

About 85% of all lung cancers are NSCLC, and they are usually less sensitive to chemotherapy than SCLC. Only 20–25% of patients present at diagnosis with early stage NSCLC and mainly undergo resection with curative intent. The vast majority present with advanced disease, and chemotherapy, targeted therapy and immunotherapy are the principal treatment strategies.

Smoking is by far the main risk factor for lung cancer [2]. Other causes include radon exposure, exposure to substances such as asbestos, nickel, chromium, beryllium, soot, and tar, a family history of lung cancer, and air pollution [3].

NSCLC is usually classified on the basis of the 8th edition of the TNM classification for non-small lung cancer, as follows: early, non metastatic disease (stages IA-IIIA tumors), locally advanced disease confined to the thoracic cavity (stage IIIB), or distant metastasis outside of the thoracic cavity (stage IV) [4].

Survival rates decrease significantly for stage I through IV as the disease progresses. The five-year survival rate for stages I, II, III and IV is 47%, 30%, 10%, and 1%, respectively [1,5].

### 1.2. Biomarkers Used in Patient Selection for Treatment with Immune Checkpoint Inhibitors

Although immune checkpoint inhibitors (ICIs) have changed the treatment paradigm for a variety of cancers, including NSCLC, not all patients respond to immunotherapy in the same way. The identification of biomarkers that are predictive of response to ICIs would help to improve the outcome of NSCLC patients. The only biomarker approved for patient selection in clinical practice is PD-L1 expression, evaluated by immunohistochemistry (IHC) [6,7,8]. This methodology is inexpensive and can be performed using standard histopathology equipment. The PD-L1/tumor proportion score is fundamental to stratify patients for first-line treatment of advanced NSCLC; the cut-off is 50% for pembrolizumab [9] and 1% for nivolumab plus ipilimumab [10]. However, while some studies [11,12,13] have reported better survival for patients with higher PD-L1 expression levels, other trials [14,15] have shown that a benefit from immunotherapy is achieved regardless of PD-L1 expression. However, inconsistencies have been observed in trials of patients enrolled with similar criteria, treated with the same inhibitor and selected using the same diagnostic antibody. Moreover, it has been seen that the type of test impacts a patient’s response to therapy and varies according to the country where it is performed [16].

The recommendation of the Food and Drug Administration (FDA) includes the use of a specific anti-PD-L1 clone, whereas European Medicines Agency (EMA) guidelines advise the use of validated IHC test. This opens up the possibility of different methods of assay validation among laboratories, i.e., the use of companion diagnostic versus non-companion diagnostic tests. However, the lack of specific guidelines could lead to discrepancies in technical and/or clinical validation procedures for PD-L1 testing.

### 1.3. Role of Tumor Mutation Burden (TMB)

#### 1.3.1. TMB Definition

TMB is defined as the total number of somatic/acquired mutations per coding area of a tumor genome (Mut/Mb). It is known that the number of mutations can vary across different tumor types. Tumors with a high mutation burden have the potential to generate a larger number of neoantigens, making them more immunogenic [17].

Non-synonymous mutations have the potential to generate neoantigens recognized by the host immune system, leading to an antitumor immune response. Mechanisms responsible for DNA mutation induction include mismatch repair (MMR) deficiency, exposure to environmental mutagens (tobacco smoke and UV light) and virus infections.

Previous studies have shown that TMB could be a potential predictive biomarker of response to ICIs [18], but problems in its detection have arisen due to various biological, methodological and economic issues. TMB is, in fact, highly dynamic and changes in primary tissue and metastasis. It may also be heterogeneous within the same tumor lesion. Another common problem is the number of cells present in small biopsies, often insufficient for the correct evaluation of TMB.

#### 1.3.2. TMB Detection

To overcome these issues, some studies have highlighted the possibility of evaluating TMB in blood. The ability to assess the tumor genome in a simple blood sample has several advantages with respect to tissue biopsy collection. The blood represents a ready-made source of diagnostic material that is less susceptible to potential sampling biases associated with tissue biopsies of a specific single site. The use of blood-based detection of DNA has increased rapidly and numerous techniques can be used to assess mutations in cell-free DNA (cfDNA), such as allele-specific PCR, digital droplet PCR and panel-based NGS [19]. The first blood-based assay (cobas EGFR Mutation Test v2, Genentech, San Francisco, CA, USA) was recently approved by the FDA for EGFR gene mutation detection. Although the main blood-based assays used for predictive biomarker detection are PCR-based, some authors have shown that NGS is also a robust approach. However, the majority of these NGS panels have a relatively limited genomic content and have not been analytically or clinically validated. Thus, although the calculation of TMB from blood has been widely promoted, robust, validated tests to measure TMB using cfDNA have yet to be developed [20].

Qiu and colleagues analyzed the performance of two plasma-based commercial TMB assays, including the effect of two different collection methods. Their results suggested that the two plasma-based TMB assays were highly correlated, and that they were also both correlated with a tissue-based TMB assay for relatively high TMB samples [21].

However, further studies are needed to confirm the reproducibility and clinical usefulness of TMB results obtained in tissue and liquid biopsies. The methodological issues are due to the fact that different platforms and cut-offs for TMB evaluation have been used and proposed.

Progenitor studies include whole-exome sequencing (WES). Campesato and colleagues concluded that cancer gene panels (CGPs) can be used to estimate mutational load and to predict clinical benefit to PD-1 blockade, with a similar accuracy to that obtained from WES [18].

To render TMB a usable biomarker for routine clinical practice, the concordance between WES and gene panel assays has been studied by several authors using different types of tests with different numbers and types of genes.

#### 1.3.3. TMB Pitfalls

##### How Many Genes Need to Be Analyzed for “a Perfect” TMB?

Different cut-offs in terms of number of mutations (e.g., 9 or 15 Mut/MB) have been proposed to ensure the highest sensitivity and specificity for the selection of immune checkpoint-responsive patients. The lack of standardization in relation to the type and number of genes to be considered affects the interpretation of results. The tests are often company sponsored and depend on the platform available in the diagnostic laboratory.

From an economic point of view, one of the reasons for the non-routine use of TMB or some large gene panels is the lack of test reimbursement. Not all laboratories and patients can afford to pay 2500 euro or 2500 dollars for a molecular test without reimbursement. The type of the chosen test depends on the country in which the patient lives.

Recently, the FDA approved the gene panel assay FoundationOne CDx12 and authorized MSK-IMPACT for the profiling of solid tumors for molecular alterations. These assays are available in the clinical setting, whereas other types of tests are only available for research purposes. These assays differ in terms of their characteristics such as enrichment approach, sequencing system, bioinformatic pipeline, number of genes and the types of mutations analyzed (e.g., synonymous and/or nonsynonymous), and recommendations for the interpretation of results.

The difficultly in standardizing a cut-off for TMB evaluation to select patients for treatment with ICIs in different tumor types may be due to differences in tumor biology and microenvironment [22]. Some findings suggest that different mutations induce a different immunological response. Neoantigens derived from frameshift mutations, insertion–deletion mutations, and mutations that influence RNA splicing are more immunogenic than non-synonymous single nucleotide mutations, which are also included in the measurement of TMB [22,23,24]. Some studies have shown that tumor immunogenicity and, consequently, response to ICIs, may be increased by a similarity between some neoantigens and antigens derived from some pathogens, but the mutations responsible for the production of these neoantigens have the same weight in TMB measurements as the other less immunogenic mutations [25]. This observation may explain the limits of using TMB as a predictive factor of the efficacy of treatment with ICIs. Moreover, mutational clonality and human leukocyte antigen (HLA) locus heterozygosity, with their associated neoantigen diversity, may play a predominant role in the development of anti-tumor immunity, but these parameters are not considered for TMB measurement [26,27]. Tumor inflammation [28], cancer stemness and intra-tumoral heterogeneity also appear to influence the immune response and the efficacy of ICIs [29,30]. The meta-analysis carried out by Lu and colleagues showed that the performance of TMB measurements in predicting the efficacy of ICIs is lower than that of other biomarkers [31]. Although several cellular components of the tumor microenvironment regulate anti-tumor immunity, their presence is not considered to be a predictive biomarker for ICIs [32].

Given that differences in both tumor types and primary resistance mechanisms can limit the usefulness of TMB scoring systems, dynamic TMB cut-offs should be considered for different tumors. For this reason, immunological features of the tumor microenvironment should be taken into account in future research to improve the decision-making process for immunotherapy [33].

#### 1.3.4. TMB in Clinical Practice

Recently, pembrolizumab was approved by the FDA for the treatment of solid tumors with high TMB. For this reason, a better definition of the biomarkers of sensitivity and resistance to immunotherapy is fundamental. Immunotherapy sequencing may play an important role in maximizing the benefit of these compounds. Cytotoxic and mutagenic chemotherapies often administered to cancer patients may increase the TMB. A tumor with an initially low TMB may develop a high TMB (TMB-H). Given the recent approval of pembrolizumab in patients with tumors with TMB ≥ 10 muts/Mb, it is expected that a higher number of patients will be treated with anti-PD-1 immunotherapy. Further studies are needed to understand whether treatment failure with anti-PD-1 therapy in earlier treatment lines impacts subsequent responses to combination immunotherapy regimens. However, recent guidelines recommend using an ICI in combination with platinum-based chemotherapy as first-line treatment for NSCLC. This is because PD-L1, albeit “special”, is not a perfect marker and there has been wide variability in its results in different studies due not only to technical problems (pre-analytical phases and immunohistochemistry methodology) but also to its intrinsic limits as a marker. For example, in a patient who lacks specific druggable driver mutations, response to a targeted drug is highly improbable, whereas in a patient with PD-L1 < 1% or with low TMB score, response to ICIs is quite probable. A predictive biomarker for immunotherapy needs to include multiple and dynamic variables that are difficult to be encompassed in a single biological factor. Currently, predictive biomarkers for targeted therapy are defined dichotomously as positive or negative (e.g., EGFR, ALK, ROS1). In the future, predictive biomarkers for ICIs should be represented by a matrix of multiple variables, e.g., TMB and tumor signatures, which could define an integrated “immunogram” [34]. Given that immunotherapy is undergoing rapid development and evolution, the fine-tuning of an integrated biomarker of sensitivity or resistance to ICIs should be considered urgent.

Great effort has made to establish guidelines to harmonize tumor TMB evaluation. Recently, Merino and colleagues, by directly comparing panel-based TMB estimates from different laboratories, characterized the theoretical variability of panel-based TMB estimates and created guidelines on TMB reporting, analytic validation requirements and reference standard alignment to maintain the consistency of TMB estimation across platforms [35].

In 2020, the European Society for Medical Oncology (ESMO) recommended the use of NGS at three levels. Specifically, routine use of NGS in tumor samples of advanced NSCLC, prostate cancer, ovarian cancer and cholangiocarcinoma is recommended. ESMO advises the use of large multigene panels over that of small panels only when the extra cost is affordable. Another recommendation concerns the use of off-label drugs, which should include genomic evaluations only if an access program has been developed and approval has been obtained at the national or regional level. Finally, an acceleration of development is recommended for drugs in clinical research centers where multigene sequencing is developed to screen patients for eligibility in clinical trials and to collect data that could be used to optimize this technology and render it usable in clinical practice. Methodological standardization is needed to introduce TMB assay into clinical practice. It is important to raise awareness of all test considerations to ensure that reliable and accurate TMB assessments can be made and compared across studies.

Given the complexity of the immune response and tumor biology, some authors have compared and combined TMB with other biomarkers to find surrogate markers of TMB capable of accurately identifying patients who are more likely to respond to immunotherapies. Jia and colleagues analyzed the transcriptome-based correlation between mutS homolog 2 (MSH2) and TMB [36] by combining three immunotherapeutic cohorts with two independent Cancer Genome Atlas (TCGA) datasets. Using multivariate analysis, the authors found that the expression of MSH2, one of the most important genes involved in the DNA mismatch repair (MMR) pathway, was associated with increased TMB. MSH2 expression also showed a significantly positive association with smoking signature, whereas an inverse association was found with MMR deficiency (MMRd) signature in lung adenocarcinoma (LUAD). A high MSH2 expression was significantly related to increased PD-L1 expression and CD8+ T cell infiltration. These findings suggest that, under these conditions, the tumor microenvironment is prominently responsive to immunotherapy. The MSH2 assessment may be less expensive, but also easier and faster, than TMB. The two biomarkers could be complementary and used to identify responders versus non-responders.

Although it has been reported that TMB does not correlate with PD-L1 expression, some authors have found that they have similar predictive capacity. TMB and PD-L1 expression could thus be incorporated into multivariable predictive models to have a greater predictive power. However, it is natural to ask whether very low TMB limits the benefit of ICIs. There is evidence to suggest that low TMB is associated with a benefit from ICIs in some tumor types such as glioblastoma. Patients with this tumor type showed favorable survival outcomes during immunotherapy despite very low TMB. Conversely, patients with high TMB have a poor response to anti-PD-1 agents. This apparent contradiction can be explained by tumor heterogeneity. The analysis of TMB from a single core biopsy may not be fully representative of the actual overall TMB. Moreover, tumor heterogeneity may be amplified by chemotherapy and radiotherapy, which can modify the mutational load in various tumor sites.

**Figure 1:**
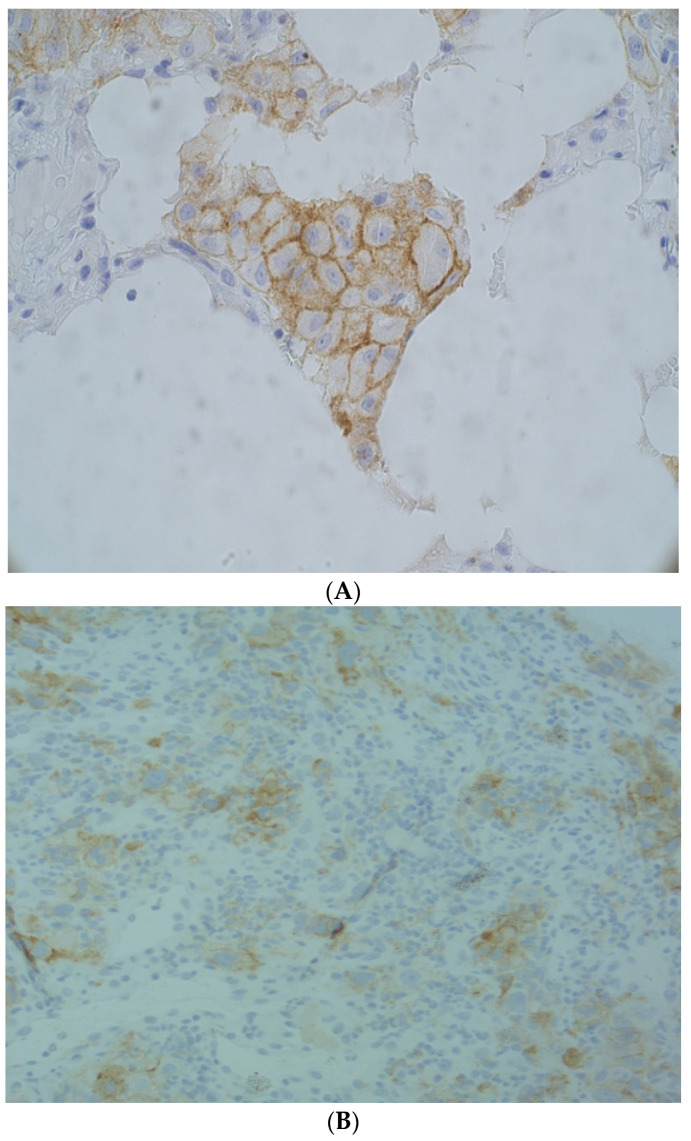
Non-small-cell lung cancer stained by immunohistochemistry with SP263 Antibody: (**A**) PD-L1-positive tumor cells (40× magnification); (**B**) PD-L1-positive tumor cells and immune cells (20× magnification).

Some patients may have high TMB and high PD-L1 or low TMB and low PD-L1, while in others these may not be concordant. In fact, in our clinical experience we also had a patient with low TMB but high PD-L1 expression. However, a biological explanation for a concordance between PD-L1 and TMB has yet to be found. In our patient, male, former light smoker, diagnosed with NSCLC, a histological sample was assessed through FoundationOne^®^ CDx, an NGS-based assay that identifies genomic findings within hundreds of cancer-related genes. We found some gene alterations (BRAF G469V; STK11 splice site 734 + 1G > T; RBM10 T852fs*28; TP53 V122fs*10), microsatellite stability (MSS) and a low TMB (3 Muts/Mb). Moreover, PD-L1 expression, evaluated as (tumor proportion score) TPS by immunohistochemistry, was 30% (Figure 1A,B). Treatment with platinum-based chemotherapy was started, but after three cycles the patient progressed and subsequently died.

As shown by Skoulidis et al., STK11 mutations are associated with negative PD-L1 expression and high TMB [37], which is the opposite of the molecular profile found in one of our patients. Skoulidis’ patient also harbored a non-V600E BRAF mutation. We know that BRAF mutations, including V600E and non-V600E, are generally associated with high PD-L1 expression, low/intermediate TMB, and MSS [38], a molecular profile similar to that seen in this case. On the basis of this observation, we can hypothesize that PD-L1, TMB and MSS status were discordant with STK11 mutation, but concordant with BRAF mutation, because BRAF mutation is an oncogene driver, which is dominant with respect to mutations in STK11, a tumor suppressor gene. Thus, although PD-L1 expression, TMB and MSS status are influenced by both tumor suppressor genes and oncogenes, an oncogene driver may dominate this influence. For this reason, the analysis of TMB per se is not sufficient as a prognostic and predictive factor because it should be considered within the whole molecular context that includes alterations in tumor suppressor genes, oncogene drivers, immune checkpoint expression, tumor mutational load, and microsatellite instability. The use of NGS assessment, like FoundationOne^®^ CDx, could be re-interpreted as a comprehensive evaluation of tumor biology and the relationship between the various molecular alterations. In patient with high TMB and concomitant high PDL1 expression, only one immune checkpoint inhibitor can be used, whereas in the event of discordance between the two biomarkers, combined treatment strategies are needed.

### 1.4. Data on Lung Cancer Trials

Within the context of some clinical trials studying the efficacy of immune checkpoint inhibitors in NSCLC patients, retrospective analyses were performed to assess the role of TMB from tumor tissue. Among these, the phase III trial CheckMate 026 studied nivolumab versus platinum-based chemotherapy in NSCLC patients with PD-L1 > 1% expression. In this study, TMB was assessed by whole exome sequencing and classified as low (0–100 mutations), medium (100–242 mutations) or high (>242 mutations). High TMB was associated with increased overall response rate (ORR) and longer progression-free survival (PFS) in patients undergoing nivolumab with respect to chemotherapy. Moreover, although TMB was not correlated with PD-L1 expression in this study, the ORR was high (75%) in patients with high TMB and PD-L1 >50% [39]. In the CheckMate 227 trial, nivolumab was also evaluated as a single agent. TMB cut-off was 13 mutations/Mb, but PFS was not improved in patients treated with nivolumab monotherapy compared to chemotherapy when both TMB and PD-L1 were high [40]. TMB was also evaluated in the POPLAR randomized phase II trial comparing second-/third-line atezolizumab with docetaxel. Improved ORR, PFS and OS were observed in patients with an increased TMB [41]. Finally, TMB was evaluated in the phase I trial CheckMate 012, which studied the combination of nivolumab and ipilimumab in untreated NSCLC patients. A higher TMB was observed in patients showing an objective response to treatment compared to those with stable or progressive disease. In this study, high TMB was defined in more than 158 mutations, which was the median value. Furthermore, in this study, TMB did not correlate with PD-L1 expression [42]. Similar results were found in the aforementioned trial, CheckMate 227, which included a comparison between nivolumab plus ipilimumab and chemotherapy. With a cut-off of 10 mutations/Mb, high TMB was associated with significantly improved ORR, one-year PFS and duration of response [40].

The development of small-cell lung cancer (SCLC) is usually the result of the inactivation of tumor suppressor genes, such as TP53, Notch, RB1 or the amplification of MYC oncogene [43]. However, SCLC cells appear to be dependent on a disruption of the DNA damage response (DDR) repair pathway. In this kind of lung cancer, a high TMB is associated with mutations in some genes of the DDR pathway, including homologous recombination, DSB, SSB, MMR and NHEJ [44]. The high TMB in SCLC can also be induced by the broad mutagenesis correlated with cigarette smoking, a well-known risk factor for SCLC. However, the association of TMB with response to immunotherapy in SCLC patients is conflicting on the basis of the findings from some clinical trials. For example, the phase I/II non-randomized trial CheckMate 032 showed an improvement in the ORR and one-year OS rate in a cohort of SCLC patients with a high TMB, mainly in those treated with the combination of nivolumab and ipilimumab [45]. Conversely, the phase III trial IMpower133 did not report an improvement in the OS of patients with high TMB treated with atezolizumab plus platinum-based chemotherapy with respect to those with low TMB receiving the same treatment [46]. This discrepancy may be due to the different assays and cut-offs used, but may also be a consequence of the addition of an anti-CTLA-4 to the anti-PD-1. For this reason, prospective studies are needed to clarify the role of TMB in patients with SCLC.

## 2. Conclusions

Despite promising results and efforts to standardize TMB calculation, it is still not routinely used in clinical practice due to the poor reproducibility of TMB results among laboratories, indicating the need for more harmonization studies. TMB detection in liquid biopsy compared to its evaluation in tissue also warrants further investigation.

FDA approval of the use of pembrolizumab in solid tumors with high TMB score opened the need to improve the definition of predictive biomarkers for treatment with ICIs. Immunotherapy should also be used at a specific point in treatment sequence of NSCLC because chemotherapy can be mutagenic and may thus induce a higher TMB score.

Although ICIs appear to be more effective in the first-line setting than in later treatment lines regardless of the TMB score, it is still not known whether this has an impact on OS. Given that PD-L1 expression and TMB score are not strictly associated with the efficacy of immunotherapy, as opposed to the presence of driver mutations (e.g., EGFR, ALK, ROS1) for targeted therapy, the search for a single biological marker should be substituted with the use of a matrix of multiple interdependent and continuous variables to select patients for immunotherapy. Some authors have proposed setting up an “immunogram” to integrate the various immune components of the tumor microenvironment. TMB and other tumor-derived or host-derived signatures could be part of this complex, multi-level predictive biomarker for immunotherapy.

## Data Availability

Not applicable.
